# Audit on Documentation and Compliance With Driver and Vehicle Licensing Agency (DVLA) Guidance for Inpatients in an Older Adult Psychiatry Ward

**DOI:** 10.1192/bjo.2026.11820

**Published:** 2026-06-30

**Authors:** Rahul Palayi, Anjana Kurungot, Nehmiea Melese

**Affiliations:** Nottinghamshire healthcare NHS foundation trust, Nottingham, United Kingdom

## Abstract

**Aims:**

To assess documentation of driving status and fitness to drive among older adult psychiatric inpatients, implement interventions, and evaluate subsequent improvement in both documentation and compliance with Driver and Vehicle Licensing Agency (DVLA) guidance.

**Methods:**

Older adult psychiatric inpatients may be unfit to drive, yet documentation of driving status is often poor, posing patient safety and medico-legal risks. A retrospective review of electronic medical records was conducted for patients discharged from a single older adult psychiatric ward over two three-month audit cycles. Data collected included documentation of driving status, presence of psychiatric diagnoses relevant to DVLA guidance, and, where relevant, whether the patients had been advised about fitness to drive. Following the initial audit, interventions were implemented, including clinician education and addition of prompts within the ward round template. The audit was then repeated as a retrospective review of records for a separate three-month period to assess the impact of these interventions.

**Results:**

In the first audit cycle, 20 patients were discharged. Driving status was documented in only 5 patients (25%), despite 18 patients (90%) having diagnoses relevant to DVLA guidance. Among those with documented driving status, only 3 patients had recorded discussion of DVLA advice, allowing adherence to be assessed. For the majority of patients, adherence could not be reliably assessed due to missing documentation, highlighting a significant gap in clinical recording and medico-legal risk.

In the re-audit cycle, 11 patients were discharged. Driving status was documented in 10 patients (90.9%), and 9 patients (81.8%) had diagnoses relevant to guidance. 7 patients were documented as not driving. Both patients documented as driving and for whom guidance applied, had evidence that DVLA advice had been discussed. Adherence could not be assessed for one patient whose driving status was not recorded. These findings indicate improved documentation and consistent discussion of driving advice following targeted interventions.

**Conclusion:**

This audit identified substantial deficiencies in baseline documentation of driving status and discussion of DVLA guidance, posing safety and medico-legal risks. Targeted interventions improved recording of driving status and consistent documentation of advice for those driving, demonstrating enhanced staff awareness and compliance with guidance. Some gaps remain, emphasizing the need for ongoing routine assessment, documentation, and staff education to ensure sustained patient safety and adherence to national guidance.

